# Supplementary material for: A Comprehensive Superposition of Viral Polymerase Structures
Source: Viruses. 2019 Aug 13;11(8):745. doi: 10.3390/v11080745 (PMC6723251; doi:10.3390/v11080745)
Supplement: Supplementary file 5 [file viruses-11-00745-s005.pdf]

Structure based alignment of picornaviral polymerases – FILE: **4-pico/sav/pico_v5.aln**

```
             1
COXA_E.pdb   GEIQWMKSNK ETGRLNINGP TRTKLEPSAF YDVFEGSKKP AVLTSKDPRL E--VDFEQAL FSKYVGNTLH EPD-----EY
COXB_A.pdb   GEIEFIESSK DAGFPVINTP SKTKLEPSVF HQVFEGNKEP AVLRSGDPRL K--ANFEEAI FSKYIGNVNT HVD-----EY
EV68_A.pdb   GEIVSNEKS- ---GVCINAP AKTKLQPSVF HQVFEGSKEP AVLNSKDPRL K--TDFEEAI FSKYTGNKIM LMD-----EY
EV71_A.pdb   GEIQWVKPNK ETGRLSINGP TRTKLEPSVF HDVFEGNKEP AVLHSKDPRL E--VDFEQAL FSKYVGNTLY EPD-----EY
POLI_A.pdb   GEIQWMRPSK EVGYPIINAP SKTKLEPSAF HYVFEGVKEP AVLTKNDPRL K--TDFEEAI FSKYVGNKIT EVD-----EY
RHIN_A.pdb   GQIQISKHVK DVGLPSIHTP TKTKLQPSVF YDIFPGSKEP AVLTEKDPRL K--VDFDSAL FSKYKGNTEC SLN-----EH
EMCV_A.pdb   GALERLPDG- ----PRIHVP RKTALRPTVA RQVFQPAFAP AVLSKFDPRT D--ADVDEVA FSKHTSNQET LP------PV
FMDV_A.pdb   GLIVDTRDVE ----ERVHVM RKTKLAPTVA HGVFNPEFGP AALSNKDPRL NEGVVLDEVI FSKHKGDTKM ---SAEDKAL

            81
COXA_E.pdb   VTQAALHYAN QLKQLDINV- -----NKMSM EEACYGTEYL EAIDLHTSAG YPYSALGVKK RDILDPITRD TTKMKFYMDK
COXB_A.pdb   MLEAVDHYAG QLATLDIST- -----EPMKL EDAVYGTEGL EALDLTTSAG YPYVALGIKK RDILSKKTKD LTKLKECMDK
EV68_A.pdb   MEEAVDHYVG CLEPLDISV- -----DPIPL ESAMYGMDGL EALDLTTSAG FPYLLQGKKK RDIFNRHTRD TTEMTKMLEK
EV71_A.pdb   IKEAALHYAN QLKQLEINT- -----SQMSM EEACYGTENL EAIDLHTSAG YPYSALGIKK RDILDPTTRD VSKMKFYMDK
POLI_A.pdb   MKEAVDHYAG QLMSLDINT- -----EQMCL EDAMYGTDGL EALDLSTSAG YPYVAMGKKK RDILNKQTRD TKEMQKLLDT
RHIN_A.pdb   IQVAVAHYSA QLATLDIDP- -----QPIAM EDSVFGMDGL EALDLNTSAG YPYVTLGIKK KDLINNKTKD ISKLKLALDK
EMCV_A.pdb   FRMVAREYAN RVFA-----L LGRDNGRLSV KQALDGLEGM DPMDKNTSPG LPYTTLG--- ---------- ----------
FMDV_A.pdb   FRRCAADYAS RLHS-----V LGTANAPLSI YEAIKGVDGL DAMEPDTAPG LPWALQG--- ---------- ----------

           161
COXA_E.pdb   YGLD------ ---------- ---------- ------LPYS TYVKDELRSL DKIRKGKSRL IEASSLNDSV YLRMTFGHLY
COXB_A.pdb   YGLN------ ---------- ---------- ------LPMV TYVKDELRSI EKVAKGKSRL IEASSLNDSV AMRQTFGNLY
EV68_A.pdb   YGVD------ ---------- ---------- ------LPFV TFVKDELRSK EKVEKGKSRL IEASSLNDSV AMRVAFGNLY
EV71_A.pdb   YGLD------ ---------- ---------- ------LPYS TYVKDELRSI DKIKKGKSRL IEASSLNDSV YLRMAFGHLY
POLI_A.pdb   YGIN------ ---------- ---------- ------LPLV TYVKDELRSK TKVEQGKSRL IEASSLNDSV AMRMAFGNLY
RHIN_A.pdb   YGVD------ ---------- ---------- ------LPMI TFLKDELRKK DKIAAGKTRV IEASSINDTI LFRTVYGNLF
EMCV_A.pdb   ----MRRTDV VDWETATLIP FAAERLEKMN NKDFSDIVYQ TFLKDELRPI EKVQAAKTRI VDVPPFEHCI LGRQLLGKFA
FMDV_A.pdb   ----KRRGAL IDFENGTVGP EVEAALKLME KREYK-FACQ TFLKDEIRPM EKVRAGKTRI VDVLPVEHIL YTRMMIGRFC

           241
COXA_E.pdb   ETFHANPGTV TGSAVGCNPD VFWSKLPILL PG-------- SLFAFDYSGY DASLSPVWFR ALEVVLREIG Y---PEEAVS
COXB_A.pdb   KTFHLNPGVV TGSAVGCDPD LFWSKIPVML DG-------- HLIAFDYSGY DASLSPVWFA CLKMILEKLG Y---THKETN
EV68_A.pdb   ATFHSNPGTA TGSAVGCDPD IFWSKIPILL DG-------- EIFAFDYTGY DASLSPVWFA CLKKVLIKLG Y---T-HQTS
EV71_A.pdb   ETFHANPGTI TGSAVGCNPD TFWSKLPILL PG-------- SLFAFDYSGY DASLSPVWFR ALELVLREIG Y---SEGAIS
POLI_A.pdb   AAFHKNPGVI TGSAVGCDPD LFWSKIPVLM EE-------- KLFAFDYTGY DASLSPAWFE ALKMVLEKIG F-GD---RVD
RHIN_A.pdb   SKFHLNPGVV TGCAVGCDPE TFWSKIPLML DG-------D CIMAFDYTNY DGSIHPIWFK ALGMVLDNLS F------NPT
EMCV_A.pdb   SKFQTQPGLE LGSAIGCDPD VHWTAF---- --GVAMQGFE RVYDVDYSNF D--------- ---------- ----------
FMDV_A.pdb   AQMHSNNGPQ IGSAVGCNPD VDWQRF---- --GTHFAQYR NVWDVDYSAF D--------- ---------- ----------

           321
COXA_E.pdb   LIEGI----- ---------- ---------- --------NH THHVYRNRTY CVLGGMPSGC SGTSIFNSMI NNIIIRTLLI
COXB_A.pdb   YIDYL----- ---------- ---------- --------CN SHHLYRDKHY FVRGGMPSGC SGTSIFNSMI NNIIIRTLML
EV68_A.pdb   FIDYL----- ---------- ---------- --------CH SVHLYKDRKY IVNGGMPSGS SGTSIFNTMI NNIIIRTLLI
EV71_A.pdb   LIEGI----- ---------- ---------- --------NH THHVYRNKTY CVLGGMPSGC SGTSIFNSMI NNIIIRALLI
POLI_A.pdb   YIDYL----- ---------- ---------- --------NH SHHLYKNKTY CVKGGMPSGC SGTSIFNSMI NNLIIRTLLL
RHIN_A.pdb   LINRL----- ---------- ---------- --------CN SKHIFKSTYY EVEGGVPSGC SGTSIFNSMI NNIIIRTLVL
EMCV_A.pdb   -----STHSV AMFRLLAEEF FSEENGFDPL VKDYLESLAI SKHAYEEKRY LITGGLPSGC AATSMLNTIM NNIIIRAGLY
FMDV_A.pdb   -----ANHCS DAMNIMFEEV FRTEFGFHPN AEWILKTLVN TEHAYENKRI TVEGGMPSGC SATSIINTIL NNIYVLYALR

           401
COXA_E.pdb   KTFKGIDLDE LHMVAYGDDV LASYPFPIDC LELAKTGKEY GLTMTP--KS PC----FN-E VTWENATFLK RGFLPDHQFP
COXB_A.pdb   KVYKGIDLDQ FRMIAYGDDV IASYPWPIDA SLLAEAGKGY GLIMTPADKG EC----FN-E VTWTNVTFLK RYFRADEQYP
EV68_A.pdb   RVYKGIDLDQ FKMIAYGDDV IASYPHKIDP ALLAEAGKHY GLVMTPADKG TS----FV-D TNWENVTFLK RYFRADDQYP
EV71_A.pdb   KTFKGIDLDE LNMVAYGDDV LASYPFPIDC LELAKTGKEY GLTMTPADKS PC----FN-E VNWDNATFLK RGFLPDEQFP
POLI_A.pdb   KTYKGIDLDH LKMIAYGDDV IASYPHEVDA SLLAQSGKDY GLTMTPADKS AT----FE-T VTWENVTFLK RFFRADEKYP
RHIN_A.pdb   DAYKHIDLDK LKIIAYGDDV IFSYKYKLDM EAIAKEGQKY GLTITPADKS SE----FK-E LDYGNVTFLK RGFRQDDKYK
EMCV_A.pdb   LTYKNFEFDD VKVLSYGDDL LVATNYQLNF DRVRTSLAKT GYKITPANKT ST----FPLE STLEDVVFLK RKFKKE---G
FMDV_A.pdb   RHYEGVELDT YTMISYGDDI VVASDYDLDF EALKPHFKSL GQTITPADK- --SDKGFVLG HSITDVTFLK RHFHMDYGTG

           481
COXA_E.pdb   FLIHPTMPMR EIHESIRWTK DARNTQDHVR SLCLLAWHNG KEEYEKFVST IRSVPIGR-A LAIPNYENLR RNWLELF---
COXB_A.pdb   FLVHPVMPMK DIHESIRWTK DPKNTQDHVR SLCLLAWHNG EHEYEEFIRK IRSVPVGR-C LTLPAFSTLR RKWLDSF---
EV68_A.pdb   FLIHPVMPMK EIHESIRWTK DPRNTQDHVR SLCYLAWHNG EEAYNEFCRK IRSVPVGR-A LTLPAYSSLR RKWLDSF---
EV71_A.pdb   FLIHPTMPMR EIHESIRWTK DARNTQDHVR SLCLLAWHNG KQEYEKFVST IRSVPVGR-A LAIPNYENLR RNWLELF---
POLI_A.pdb   FLIHPVMPMK EIHESIRWTK DPRNTQDHVR SLCLLAWHNG EEEYNKFLAK IRSVPIGR-A LDLPEYSTLY DRWLDSF---
RHIN_A.pdb   FLIHPTFPVE EIYESIRWTK KPSQMQEHVL SLCHLMWHNG PEIYKDFETK IRSVSAGR-A LYIPPYELLR HEWYEKF---
EMCV_A.pdb   PLYRPVMNRE ALEAMLSYYR P-GTLSEKLT SITMLAVHSG KQEYDRLFAP FREV-----G VIVPTFESVE YRWRSLFW--
FMDV_A.pdb   -FYKPVMASK TLEAILSFAR R-GTIQEKLI SVAGLAVHSG PDEYRRLFEP FQ------GL FEIPSYRSLY LRWVNAVCGD

           561
COXA_E.pdb   -------
COXB_A.pdb   -------
EV68_A.pdb   -------
EV71_A.pdb   -------
POLI_A.pdb   -------
RHIN_A.pdb   -------
EMCV_A.pdb   -------
FMDV_A.pdb   AAALEHH
```

Note that sequences are derived from the PDB coordinate files and reflect any mutations present in the reported structures.
